# Neuroprotective potential of *Mucuna pruriens* in cerebral ischemia: Evidence from animal models and implications for translational neuropharmacology

**DOI:** 10.14202/vetworld.2026.1691-1706

**Published:** 2026-04-28

**Authors:** Vanishri Sunil Nayak, Karkala Sreedhara Ranganath Pai, Bukkambudhi Virupakshamurthy Murlimanju, Sunil Sanjiva Nayak, Suhani Sumalatha, Mamatha Tonse

**Affiliations:** 1 Department of Anatomy, Kasturba Medical College, Manipal Academy of Higher Education, Manipal, India; 2 Department of Pharmacology, Manipal College of Pharmaceutical Sciences, Manipal Academy of Higher Education, Manipal, India; 3 Department of Anatomy, Kasturba Medical College Mangalore, Manipal Academy of Higher Education, Manipal, India.; 4 Department of Oral and Maxillofacial Surgery, Manipal College of Dental Sciences, Manipal Academy of Higher Education, Manipal, India

**Keywords:** Cerebral ischemia, Ischemic stroke, Mucuna, Neuroprotective agents

## Abstract

Cerebral ischemia is a major neurological condition that contributes significantly to global morbidity and mortality. It occurs due to reduced blood supply to the brain, leading to neuronal injury and death. The pathophysiology of cerebral ischemia is complex and involves oxidative stress, neuroinflammation, excitotoxicity, mitochondrial dysfunction, and apoptosis. Despite advances in medical management, effective neuroprotective therapies remain limited, creating a need to explore alternative approaches. Mucuna pruriens is a medicinal plant widely used in traditional medicine, known for its rich content of bioactive compounds, including levodopa, flavonoids, alkaloids, and phenolic antioxidants. These compounds are associated with antioxidant, anti-inflammatory, and neuroprotective properties. This review summarizes the available evidence from animal model studies on the role of *M. pruriens* in the management of cerebral ischemia. The literature indicates that *M. pruriens* can reduce oxidative stress by enhancing antioxidant enzyme activities, including superoxide dismutase, catalase, and glutathione peroxidase, and by decreasing lipid peroxidation. Histopathological findings suggest that it helps preserve neuronal structure, particularly in vulnerable brain regions such as the hippocampus. Behavioral studies also demonstrate improvements in motor function, coordination, and cognitive performance following treatment with *M. pruriens*. These effects are mainly attributed to its ability to scavenge free radicals, modulate neurotransmitter levels, and regulate inflammatory pathways. Although the findings from animal studies are promising, variations in experimental design, dosage, and extraction methods limit the consistency of results. In addition, the lack of standardized formulations and clinical evidence restricts its direct application in human patients. In conclusion, *M. pruriens* shows potential as a natural neuroprotective agent in cerebral ischemia. However, further well-designed experimental and clinical studies are required to establish its safety, efficacy, and standardized therapeutic use.

## INTRODUCTION

In 2021, 7.8 million people were affected by ischemic stroke globally, and 3.6 million people died during this period [1, 2]. Ischemic stroke comprises approximately 80% of all strokes and occurs because of reduced blood supply to the brain, causing damage to and death of brain tissue. There is no effective treatment for ischemic brain damage, possibly because of complex events that include excitotoxicity, loss of calcium homeostasis, oxidative stress, inflammation, lipid peroxidation, and apoptosis [3, 4], leading to neuronal death. Apoptosis is considered one of the critical factors responsible for postischemic cell death [5]. In global cerebral ischemia, the central part of the ischemic region does not receive any blood supply, but a small amount of blood can still reach this area through collateral circulation. The best example of this is middle cerebral artery occlusion-induced ischemia [6]. Reactive oxygen species (ROS) are generated after ischemia, leading to oxidative stress, neuronal death, and brain damage [7]. Newly formed free radicals extract an electron from another molecule, thus producing a chain reaction [8]. The synthesis of antioxidants balances the continuous production of oxidants. An imbalance between reactive oxygen metabolite production and antioxidant defenses results in oxidative stress, as shown in previous studies [9]. The accumulation of hydrogen peroxide impairs mitochondrial function [10], which persists for a longer duration after reperfusion and induces neuronal damage. There is a reduction in superoxide dismutase (SOD) and catalase (CAT) activity after ischemic injury. Apoptosis and necrosis are the two types of cellular death that occur after ischemia. In the core region, necrosis is more common, whereas neuronal death in the penumbral region is predominated by apoptosis [11]. Ischemia involves dysfunction of the hippocampal formation, neocortex, and Purkinje cells of the cerebellum. The hippocampus plays a major role in learning and memory. Compared with the CA3 region and dentate gyrus, the hippocampal CA1 region is more vulnerable to ischemic insult, and damage to this region is greater [12].

Fluoride, along with small amounts of aluminum, affects calcium influx and mobilization, neurotransmission, cell growth, differentiation, and cytoskeletal proteins [13]. Aluminum fluoride can affect learning and memory [14]. Various factors are involved, including synaptic plasticity because of the inhibition of long-term potentiation through the phosphoinositide 3-kinase–protein kinase B–mammalian target of rapamycin and brain-derived neurotrophic factor–tropomyosin receptor kinase B pathways [15, 16]. Disruption of the balance between mitochondrial fission and fusion leads to mitochondrial dysfunction in ischemic stroke [17]. Together, activation of toll-like receptor 4, increased ROS, and neuroinflammation lead to oxidative stress [18]. Inhibition of the histone demethylase PHF8 and decreased brain-derived neurotrophic factor eventually lead to epigenetic modifications [19]. All these mechanisms of action affect long-term memory [20].

Cerebral ischemia is a major neurological condition that causes morbidity, disability, and mortality worldwide. This condition requires immediate attention and emergency treatment; however, despite the best available advanced investigations and management methods, there are issues such as a lack of early diagnosis, time limitations in administering treatment, and unclear pathophysiology and adverse effects, which include oxidative stress, neuroinflammation, and apoptosis. There is a need for alternative drugs that can exert neuroprotective effects. *Mucuna pruriens* is a medicinal plant used in traditional medicine that is rich in compounds such as flavonoids, alkaloids, levodopa, and phenolic antioxidants. The significance of this review article is that it adds to the existing knowledge by bridging the gap in understanding traditional medicine. This study offers insight into the use of animal models for evaluating cerebral ischemia and its management. This basic translational research is important for highlighting the therapeutic potential of *M. pruriens* as an adjuvant and a potential neuroprotective drug. This review will motivate the scientific community to conduct original studies on phytochemicals and their role in cerebral ischemia. The future implications of this subject include clinical research involving *M. pruriens* as a neuroprotective agent in ischemic stroke and other ischemia-associated neurological disorders. There is a need to further explore the etiopathogenesis, biomarkers, and suitable treatments available to prevent the global burden of cerebral ischemia. Overall, this study is significant in motivating animal model research in clinical neuroscience and stroke-related research.

There is no universally accepted benchmarking of phytochemical profiles and pharmacological activity in *M. pruriens*. There is a knowledge gap, as few studies have reported the beneficial effects of *M. pruriens*; however, the findings are not statistically significant, indicating a need for more rigorous research [21]. The differences in opinion among studies may be due to variability in study design, *M. pruriens* dose, and mode of administration, as *well as* in the experimental animal model. The variability in results may also be due to differences in experimental models, dosages, and the specific neurodegenerative conditions being studied [21, 22]. In this context, this narrative review critically presents emerging evidence of the neuroprotective, antioxidant, and anti-inflammatory efficacy of *M. pruriens*, providing insight into translational research in stroke and phytotherapy.

The present review aims to provide a comprehensive and critical synthesis of the available preclinical evidence on the neuroprotective potential of *M. pruriens* in cerebral ischemia, with a specific focus on animal model–based investigations. This review systematically integrates findings on oxidative stress modulation, neuroinflammatory regulation, mitochondrial protection, and antiapoptotic mechanisms associated with *M. pruriens* administration. Furthermore, it seeks to evaluate the consistency of biochemical, histopathological, and behavioral outcomes reported across different experimental ischemia models, including variations in extraction methods, dosage regimens, and routes of administration. In addition, this review aims to elucidate the mechanistic pathways underlying the neuroprotective actions of key phytoconstituents, particularly levodopa, flavonoids, and phenolic compounds, in mitigating ischemia-induced neuronal damage. A critical objective is to identify gaps and inconsistencies in the current literature, including a lack of standardized formulations, limited dose-optimization studies, and variability in experimental designs, which hinder translational applicability. Moreover, this review aims to bridge the gap between experimental findings and clinical relevance by discussing the challenges of translating animal model outcomes into human therapeutic strategies. Ultimately, the review aims to highlight the potential of *M. pruriens* as a multifunctional, phytochemical-based neuroprotective agent and to propose future research directions focusing on standardization, bioavailability enhancement, and clinical validation for its effective integration into stroke management paradigms

## REVIEW METHODOLOGY

The methodology for this narrative review was designed to include a comprehensive and critical overview of the literature. The results of the comparative analysis of animal model studies of *M. pruriens* on the central nervous system, including the types of models used, extraction types of *M. pruriens*, biochemical outcome markers, behavioral observations, and relative efficacy [21–26], are summarized in Table 1.

**Table 1:** Comparative analysis of animal model studies of *Mucuna pruriens* on the central nervous system among various studies.

Authors	Animal	Mode of experimental model	Extract of *M. pruriens*	Biochemical markers studied	Histopathological observation	Behavioral analysis
Chandran et al. [21]	Wistar albino rat	Spinal cord injury model	Ethanolic	Malondialdehyde, superoxide dismutase (SOD), catalase (CAT)	Decrease in neuronal death, axon sprouting, and glial scarring	No significant improvement
Bhosle and Wadher [22]	Wistar albino rat	Chemical ischemia-induced by 3-nitropropionic acid	Ethanolic	SOD, CAT, glutathione peroxidase (GPx)	Preservation of neuronal integrity	Improved motor function
Nayak et al. [23]	Wistar albino rat	Bilateral common carotid artery occlusion	Methanolic	SOD, CAT, GPx	Neuro-restoration, increased number of viable neurons	Improved locomotion, coordination of movements, and spatial learning
Dogra et al. [24]	Zebrafish	Rotenone-induced neurodegeneration	Chemically standardized	CAT, glutathione-S-transferase, acetylcholinesterase, butyrylcholinesterase	Decrease in inflammation and demyelination, reduction in focal necrosis in parts of the brain	Regaining of interest and cognition
Yadav et al. [25]	Swiss albino mice	1-Methyl-4-phenyl-1,2,3,6-tetrahydropyridine-induced Parkinson’s	Ethanolic	Dopamine, 3,4-dihydroxyphenylacetic acid, homovanillic acid	Increase in the number of glial fibrillary acidic protein-positive neurons and astrocytes, recovery of tyrosine hydroxylase-positive neurons	Improved neurobehavioral performance
Manyam et al. [26]	Sprague–Dawley rat	6-Hydroxydopamine-lesioned Parkinson’s model	Powdered seed, nonextracted form	Mitochondrial complex-I	Restoration of levodopa, dopamine, norepinephrine, and serotonin in the substantia nigra	Decreased symptoms of Parkinsonism

### Literature search strategy

The search was performed through electronic databases such as PubMed, Scopus, Web of Science, and Google Scholar. Studies published between 2000 and 2026 were given priority. The Medical Subject Headings browser and keyword search were used. The key search terms included “cerebral ischemia,” “*Mucuna pruriens*,” “oxidative stress,” “animal model,” and “neuroprotection.”

### Article selection criteria

Initially, articles were selected based on their titles and abstracts. This was subsequently followed by downloading the full texts to read the manuscripts completely.

### Inclusion criteria

In animal model studies of cerebral ischemia, studies evaluating any extract of *M. pruriens* and reporting biochemical, histopathological, and behavioral outcomes were included. Peer-reviewed scientific journal publications, including original animal model research, original clinical research, systematic review articles, and narrative reviews published in the English language, were the inclusion criteria for this study.

### Exclusion criteria

Editorials, nonischemic models, non-extraction studies of *M. pruriens*, *in vitro*-only studies, articles without accessible full text, conference proceedings, letters to the editor, commentaries, non-English-language publications, articles with insufficient methodological clarity, and duplicate data were excluded from the present study.

### Screening procedure

Three authors reviewed the articles included in the literature search.

### Complexity of ischemic brain injury

**Figure 1:**
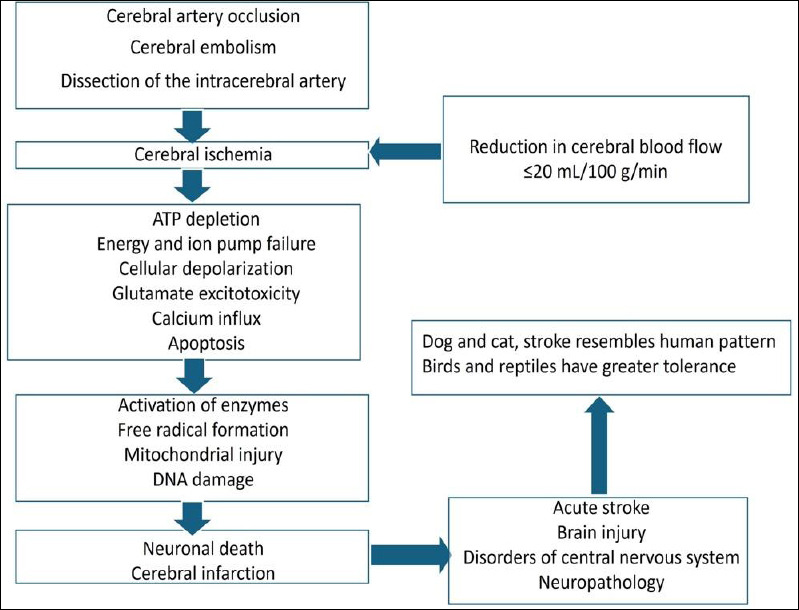
Schematic diagram showing the pathophysiology of cerebral ischemia [3, 5, 7, 8, 11, 23].

Ischemic brain injury involves complex biochemical, molecular, and cellular mechanisms. The cascade of events includes necrosis, apoptosis, the autoimmune response, and neuroplasticity [27]. Age-based variation has been reported to affect neuroprotection and ischemic outcomes [28]. Increased levels of neurotransmitters, including glutamate, contribute to excitation of receptors and subsequent neuronal injury. Disturbance in calcium ion homeostasis aggravates cell injury [29]. Astrocytic and microglial activation, along with infiltration of neutrophils and lymphocytes, leads to amplification of the inflammatory response [30]. The pathophysiology of cerebral ischemia is represented in Figure 1 [3, 5, 7, 8, 11, 23].

Research using animal models of ischemia suggests that reactive gliosis results from a response to altered perfusion. Changes in blood flow lead to glial activation and subsequent gliosis [31]. However, when chronic reactive gliosis occurs, neuronal recovery and regeneration are inhibited. Chronic astrogliosis is associated with the formation of glial scarring, which creates physical and molecular barriers that impede axon regeneration and functional recovery [32]. Microglial activation also triggers astrogliosis through the release of cytokines, which can exacerbate astrocytes’ reactive state, furthering neuronal injury.

### Ischemic cascade

Immediately after the reduction in blood flow, oxygen and glucose levels decrease, further reducing adenosine triphosphate and energy-dependent processes [33]. Chronic cerebral ischemia occurs because of long-term reduction in cerebral blood flow caused by conditions such as hypertension and atherosclerosis, resulting in impaired cognition and dementia. Ionic imbalance occurs with the influx of sodium and calcium into cells and the involvement of the mitochondrial permeability transition pore. Nitric oxide and peroxynitrite also play important roles in the etiopathogenesis of ischemia–reperfusion injury.

### Reperfusion injury in cerebral ischemia

Restoration of the blood supply can paradoxically aggravate injury through oxidative stress, inflammation, and disruption of the blood–brain barrier [30]. Leukocytic infiltration and activation of platelets and the complement system play instrumental roles [34]. Ischemia followed by reperfusion can affect mitochondria, leading to dysfunction. Injury to the mitochondrial complex and oxidative modifications are crucial aspects of the pathogenesis of ischemic neuronal injury [35]. The NLRP3 inflammasome and the Nrf2–Keap1 pathway are important during the management of reperfusion injury.

### Autophagy and the neuroinflammatory cascade

Autophagy impairment eventually leads to the accumulation of damaged organelles and protein misfolding and aggregation [36]. The proinflammatory (M1) and anti-inflammatory (M2) phenotypes of microglial polarization play crucial roles in neuroinflammation [30]. Release of chemokines and inflammatory cytokines further contributes to mediation of neuronal injury and disruption of the blood–brain barrier. The LC3-II, Beclin-1, and p62 pathways, crosstalk between autophagy and apoptosis, M1/M2 ratios, and specific microglial marker TLR4/NF-κB pathways are also important. Rapamycin and 5-aminoimidazole-4-carboxamide ribonucleotide are autophagy activators that can increase neuronal viability, reduce lactate dehydrogenase leakage, and alleviate apoptosis in oxygen–glucose deprivation/reoxygenation models by activating the adenosine monophosphate-activated protein kinase/DDiT4/mammalian target of rapamycin axis [37].

### Nuclear factor erythroid 2-related factor 2 (Nrf2)

Nrf2 is a regulator of the antioxidant response at the cellular level and activates the expression of proteins and enzymes, which help neutralize ROS-induced ischemia–reperfusion injury. Nrf2 helps maintain mitochondrial function, biogenesis, and integrity, which are essential for neuronal survival during ischemia. Its activation can inhibit iron-dependent lipid peroxidation in the context of cerebral ischemia [38, 39].

### Cerebral ischemia in an animal model

In the global ischemic model, there is complete disruption of blood flow to the brain, leading to necrosis of the cerebral region. The methods used to achieve global cerebral ischemia include increasing intracranial pressure, occlusion of major arteries, and cervical compression [40]. However, a straightforward method for inducing ischemia is decapitation, which is performed after approval from the institutional animal ethics committee. This method was used long ago in small animals to elucidate the biochemical mechanism and global ischemic pathways involved. In the global cerebral ischemic model, any artery supplying the cerebral region can be occluded. The best example is middle cerebral artery occlusion in small-animal or large-animal model studies [41]. The middle cerebral artery occlusion model is widely used because it simulates thromboembolic stroke. Ischemic models can be generated in both small and large animals. Although many drawbacks exist in using animal models to study ischemia, these models are still in use.

There may be species-specific variations in neuroanatomy and neurophysiology, including responses to ischemia. The size and structure of the brain, vasculature, neuroprotection, and metabolic responses can vary among rat models, larger animals, and humans. Induction of cerebral ischemia by occluding the middle cerebral artery can produce variable results because of differences in surgical techniques, methods of anesthesia, and postoperative care. While animal model studies can yield good results, variable outcomes can cause difficulty in interpretation of the findings. Factors such as the genetic background of the animals can affect the severity of ischemic events and the recovery phase. Therefore, the findings of animal model research cannot be generalized. However, physiological control can be achieved in these models. With the help of these models, injury, its mechanism, and its neuroprotective effects can be better understood. These phases can be challenging to study in humans during ischemia because of the varied causes, manifestations, and ischemic sites. However, studying the mechanism of injury as well as neuroprotection is highly beneficial.

Nayak et al. [23] induced cerebral ischemia through bilateral carotid artery occlusion and thereby created oxidative stress. Ischemia results in a decline in memory and learning abilities; later, locomotor activity was assessed using an actophotometer, and motor coordination was assessed using the rotarod test. This was supported by assessment of cerebral infarction on the basis of histopathological findings. This was further supported by biochemical evidence of elevated lipid peroxidase (LPO) levels and depletion of SOD, CAT, reduced glutathione (GSH), and total thiols in the ischemic group. They also chemically induced ischemia by inducing oxidative stress through the administration of aluminum fluoride in drinking water for 7 days. There were decreased levels of SOD, CAT, and glutathione peroxidase (GPx), and elevated levels of oxidative stress markers, including malondialdehyde (MDA) and ROS.

### Increased intracranial pressure (ICP) and cerebral ischemia

The standard ICP threshold is more than 20 mmHg, and it can be measured with modern ICP monitoring devices, which may be invasive. Increased ICP can result from space-occupying lesions, cerebral edema, and hematomas. This reduces cerebral perfusion pressure and cerebral blood flow, leading to global ischemia and hypoxia. It causes mechanical distortion and compression of the brain, exacerbating ischemia [42]. A reduction in cerebral blood flow can lead to metabolic dysfunction and secondary brain injury. Research has suggested a significant decrease in cortical blood flow and velocity because of increased ICP [43]. In animal models of traumatic brain injury, increased ICP leads to increased neuronal injury and behavioral changes, even without global ischemia. This finding indicates that even subischemic increases in ICP can cause significant deficits [44].

### Experimental induction of increased ICP in animal models

Rodents, canines, pigs, and nonhuman primates have been used as animal models to induce increased ICP. Conditions such as an acute subdural hematoma cause increased ICP and exacerbation because of the mass effect of the hematoma. However, experimental animal studies often involve the infusion of artificial lumbar cerebrospinal fluid to increase ICP [43, 45]. In a Japanese study, autologous arterial blood was infused into the brainstem and internal capsule to increase ICP in white rabbits [46]. In another study, a Fogarty balloon catheter was inserted and inflated to simulate a space-occupying lesion and increased ICP [47].

Injection of autologous blood into the midbrain, internal capsule, and related sites can cause hemorrhage and increased ICP. Injection of saline into the lateral ventricle or brain can also cause increased ICP. In pigs, this method involves monitoring the optic nerve sheath diameter by ultrasound, which is an indirect indicator of increased ICP [48]. Direct measurement of ICP using transducers placed in the brain parenchyma or lateral ventricle is accurate but technically difficult [48].

Institutional animal ethics committee approval is mandatory to induce increased ICP in animals. It is also suggested that the induction and measurement of increased ICP can be complex due to the need for specialized equipment and expertise, potentially leading to variable results.

### Induction of cerebral ischemia in animals by occlusion of arteries

Cerebral ischemia can be experimentally induced through various arterial occlusions. The middle cerebral artery can be occluded by electrocoagulation through the application of an electric current to coagulate the artery. Devices such as filaments can be used and adjusted to create either permanent or transient occlusions of the middle cerebral artery. Application of the endothelin-1 peptide also induces vasospasm, leading to ischemia [49]. The common carotid artery can also be occluded to induce ischemia. Occlusion of the artery leads to decreased nutrition, reduced energy production, an acidic environment, and resultant cell death [50]. This method requires strict aseptic precautions and physiological monitoring under anesthesia. The limitation of this method is the presence of collateral circulation, which can significantly influence the outcome.

Middle cerebral artery occlusion models are commonly used in stroke research to simulate ischemic conditions in the brain. These models can be categorized into permanent and transient occlusion models. Permanent occlusion is usually induced by electrocoagulation, which permanently blocks the artery without reperfusion. Transient occlusion involves temporary occlusion of the artery with an intraluminal suture, followed by reperfusion after a period ranging from 30 min to 2 h. The Longa technique involves generating a focal ischemia model by occluding the right middle cerebral artery. The animals were excluded if a subarachnoid hemorrhage occurred during middle cerebral artery occlusion [51].

### Treatment of cerebral ischemia in an animal model

Given that ROS play a significant role in cell death following ischemic injury, several studies have been conducted to assess their neuroprotective effects. Modulation of hypoxia-inducible factor alpha has also been shown to increase endothelial nitric oxide synthetase and enhance cerebral blood supply in animal models of arterial occlusion [52]. Although this concept has shown positive results in animal models, in human trials these findings have not been as robust. This concept is successful in cardiovascular surgery but not in neurological disorders. The systematic use of imaging in ischemia and its management will allow us to understand the exact phenomenon occurring in the brain [53]. Therefore, there is a need to use combined approaches in ischemic conditions, including neuroprotective and vasoprotective agents, together with revascularization treatments.

The search for a natural neuroprotective substance with minimal side effects has attracted increasing attention. Although many plants have been examined to date, only a few have been studied thoroughly. Various compounds with antioxidant properties have been studied and tested to assess their endogenous antioxidant effects. Free radical scavengers and antioxidants are commonly used to mitigate the harmful effects of oxygen-free radicals during ischemia. There are potential clinical benefits, including reduced histopathological, biochemical, and oxidative metabolic damage, as well as improved neurological outcomes. A comparison of various drugs used to manage cerebral ischemia in animal models is presented in Table 2 [54–61].

**Table 2:** Comparison of various drugs used in the management of cerebral ischemia in animal models.

Drug	Class	Mechanism of action	Experimental model	Neuroprotective effects	Dose	Translational status
Dizocilpine	N-methyl-D-aspartate receptor antagonist	Noncompetitive blockade of N-methyl-D-aspartate receptors → decreased Ca²⁺ influx → decreased excitotoxic neuronal death	Middle cerebral artery occlusion-induced global ischemia in rat, mouse, and cat	Decreased infarct volume, decreased neuronal death, improved motor and cognitive outcomes	Pre- or early postischemia (0–2 h); 0.1–5 mg/kg	Strong preclinical efficacy but failed clinical translation because of psychotomimetic and neurotoxic side effects [54]
Edaravone	Free radical scavenger	Scavenges ROS, activates Nrf2/antioxidant response element pathway, inhibits lipid peroxidation, ferroptosis, and apoptosis	Middle cerebral artery occlusion-induced global ischemia in rat	Decreased infarct size, decreased oxidative stress, decreased inflammation, increased brain-derived neurotrophic factor, improved neurological scores	Postischemia (≤6 h); 3–30 mg/kg	Clinically approved in Japan; strong alignment between animal and clinical data [55]
Edaravone–dexborneol	Antioxidant + anti-inflammatory combination	Inhibits oxidative stress, NF-κB/NLRP3, ferroptosis, and pyroptosis; enhances blood–brain barrier integrity	Middle cerebral artery occlusion-induced global ischemia in rat	Decreased infarct volume, increased cerebral blood flow, decreased neuroinflammation, improved cognition	Immediate to delayed postischemia; 0.375–15 mg/kg	Improved efficacy over edaravone alone; high translational potential [56]
Nimodipine	L-type Ca²⁺ channel blocker	Blocks voltage-gated Ca²⁺ channels, causes vasodilation, and decreases ischemic acidosis	Middle cerebral artery-induced hypertensive rat models	Decreased infarct size, improved neurological outcome	Pre- or early postischemia; continuous infusion or 1–20 mg/kg	Mixed animal results; failed large clinical trials in stroke [57]
Minocycline	Tetracycline	Anti-inflammatory (decreased microglial activation), anti-apoptotic (decreased caspase-3), decreased high-mobility group box 1, NF-κB	Middle cerebral artery occlusion-induced global ischemia in rat, mouse, and cat	Decreased infarct size, decreased apoptosis, improved long-term functional recovery	30 min–24 h postischemia; 10–90 mg/kg	Excellent blood–brain barrier penetration; safe in humans but modest efficacy [58]
Memantine	N-methyl-D-aspartate receptor antagonist	Partial N-methyl-D-aspartate blockade limits excitotoxicity while preserving physiological signaling	Middle cerebral artery-induced hypertensive rat models	Decreased neuronal loss, improved cognitive outcome	Early postischemia; variable dosing	Better tolerated than dizocilpine; limited stroke-specific data [59]
Carvacrol	Natural monoterpenoid phenol	Antioxidant, anti-inflammatory, anti-apoptotic; inhibits transient receptor potential melastatin 7 channels	Ischemia and hypoxia models in rat and mice	Decreased oxidative stress, decreased neuroinflammation, decreased apoptosis	Pre- or early postischemia; variable doses	Emerging natural neuroprotectant, preclinical stage [60]
Tissue plasminogen activator	Thrombolytic	Converts plasminogen to plasmin, resulting in clot lysis	Thromboembolic stroke models	Restores cerebral blood flow, reduces infarct size, with hemorrhage risk	≤3–4.5 h postischemia	Gold standard in clinics; often combined with neuroprotectants in animals [61]

### Herbal products as phytochemicals

Herbal products exhibit high variability in their medicinal value, particularly in antioxidant properties. This may be due to differences in species, the part of the plant used, climate conditions, and availability of these plants [62, 63]. Characterization and standardization of herbal products are based on various chemical constituents known as phytoconstituents. These phytoconstituents naturally occur in medicinal plants, leaves, seeds, vegetables, and roots and are responsible for multiple medicinal and pharmaceutical properties. They are classified into primary compounds, such as sugars and fats, and secondary compounds, such as alkaloids, tannins, terpenoids, phenols, and glycosides. These secondary compounds and other plant-derived chemical entities, such as Rheo discolor, are responsible for their medicinal value [64]. They have high contents of phenols (48.41 mg/1 g), flavonoids (43 mg/1 g), and tannins (44.93 mg/1 g). An earlier report found that phenols have strong antioxidant properties and reduce oxidative stress in the brain [65]. Flavonoids have been explored for their ability to prevent oxidative stress caused by ischemia–reperfusion. Tannins have also been reported to possess antioxidant and free-radical-scavenging activities [66]. These natural medicines are successful in preclinical studies because they are neuroprotective, ameliorating protein aggregation, oxidative stress, and neuroinflammation [67]. A comparison of the antioxidant content and strength of *M. pruriens* with those of other herbs is presented in Table 3 [68, 69].

**Table 3:** Comparison of the antioxidant content and strength of *Mucuna pruriens* with those of other herbs.

Herb	Antioxidant content	Relative strength
Withania somnifera (ashwagandha, roots/leaves)	Withanolides, flavonoids, and phenolics	Comparable to or slightly lower than *M. pruriens* [68]
Curcuma longa (turmeric, rhizome)	Curcuminoids and phenolic acids	Stronger than *M. pruriens* [69]
Ocimum sanctum (tulsi, leaves)	Eugenol, rosmarinic acid, and flavonoids	Comparable to or slightly higher than *M. pruriens*
Moringa oleifera (leaves)	Quercetin, chlorogenic acid, and vitamins	Much stronger than *M. pruriens*
Camellia sinensis (green tea, leaves)	Catechins	Much stronger than *M. pruriens*

### M. pruriens

Medicinal plants are gifts for fighting disease and death. Although many plants have been examined to date, only a handful have been studied thoroughly. The *M. pruriens* plant is also known as Kapikacchu, Atmagupta, and Naayi songe. This plant is easily digestible, has a high protein content, and is native to tropical regions of Africa, India, the Caribbean, and China. Its flowers may be white or purple. The pods have loose brownish hairs, which cause severe itching on contact with skin. The plant has black or brown beans. Currently, this plant is consumed in some countries because its pods can be used as a vegetable substitute for humans. In some parts of the world, its seeds are roasted and ground into a powder for use as a coffee substitute, such as Nescafé [70].

### Functional components of *M. pruriens*

*M. pruriens* seeds contain approximately 27% protein and minerals. The antiphysiological and toxic properties of some *M. pruriens* species are due to the presence of amino acids, polyphenols, phytates, trypsin inhibitors, cyanogenic glycosides, oligosaccharides, lectins, saponins, and alkaloids. Polyphenols bind with proteins and thus decrease their digestibility [71]. In addition to levodopa, *M. pruriens* seeds also contain tryptamine and 5-hydroxytryptamine.

### Active components identified in *M. pruriens*

Its primary active compounds include levodopa, which is present at the highest level in its seeds, ranging from 0.2% to 7.30% of dry weight [72]. Its methanolic extracts yielded 3β-hydroxy-5α-cholanic acid acetate, 3,5,7,4-tetrahydroxy-6-methoxyflavone, and ethyl 2-amino-5-hydroxy-3,6,6-trimethyl heptonate. Other bioactive compounds, such as medicarpin and parvisoflavone B, have been identified as β-glucosidase inhibitors [73]. Ursolic acid and betulinic acid, which exhibit neuroprotective activities similar to those of levodopa [74], have also been detected. The medicinal properties of *M. pruriens* also include the production of alkaloids, including prurienidine, prurienine, and prurieninine [75]. (Z)-Triacont-5,7,9-triene, (Z)-docos-2,4,6-trien-1,8-diol, and (Z)-docos-5-en-1-oic acid are its lipid derivatives. Its secondary metabolites, such as flavonoids, tannins, saponins, steroids, glycosides, and terpenoids, have antimicrobial, antioxidant, and anti-inflammatory properties [76]. However, its antioxidant properties are affected mainly by phenols and flavonoids. The phytochemical profiles of *M. pruriens* are presented in Table 4 [77–80].

**Table 4:** Phytochemical profile of *Mucuna pruriens*.

Phytochemical	Compound	Plant parts	Major pharmacological relevance
Non-protein amino acids	Levodopa	Seeds, roots, leaves	Dopamine precursor; anti-Parkinson’s activity; neuroprotection; antioxidant effects [77]
Alkaloids	Mucunine, mucunadine, prurienine, nicotine	Seeds, leaves	Central nervous system activity; antimicrobial; contribution to neuropharmacology [78]
Indole amines	Serotonin, tryptamine	Pods, hairs, seeds	Neurotransmission; role in neuromodulation
Flavonoids	Quercetin, kaempferol, rutin	Leaves, seeds	Antioxidant, anti-inflammatory [79]
Phenolic compounds	Gallic acid, caffeic acid, ferulic acid	Seeds, leaves	Strong antioxidant activity; cytoprotective effects
Tannins	Condensed and hydrolyzable tannins	Seeds, leaves	Antimicrobial, antioxidant, protein-binding properties
Saponins	Triterpenoid saponins	Seeds	Immunomodulatory effects [77]
Sterols/triterpenes	β-Sitosterol, stigmasterol	Seeds, leaves	Anti-inflammatory
Proteins and peptides	Lectins, protease inhibitors	Seeds	Immune modulation [77]
Other bioactives	Amino acids, nucleosides, vitamins, phenolic derivatives	Seeds	Antioxidant, cytoprotective [80]

### Mechanism of action of the active components of *M. pruriens*

Levodopa reportedly enhances the ability to scavenge free radicals and reduce oxidative damage in cerebral ischemia [74]. It can cross the blood–brain barrier and restore dopaminergic tone. Flavonoids, polyphenols, and tannins scavenge nitric oxide, hydroxyl radicals, and superoxide. The levels of antioxidant enzymes, including CAT, SOD, and GPx, are increased, whereas MDA and lipid peroxidation are decreased. β-Sitosterol acts as a neuroprotective agent against cerebrovascular diseases by downregulating N-methyl-D-aspartate receptor gene expression and decreasing calcium influx, which prevents excitotoxic neuronal death [67]. β-Sitosterol is also involved in the suppression of ischemia-induced tau hyperphosphorylation and preservation of the integrity and stability of axons and microtubules.

### Traditional uses of *M. pruriens*

*M. pruriens* is a traditional Indian medicinal plant that has been widely used in Ayurveda since the Vedic period. The plant is commonly known as Kapikacchu in Sanskrit and is used primarily for Parkinson’s disease and other nervous disorders, such as depression [81], and for arthritis, as well as for its aphrodisiac properties. In an animal model study with rotenone-intoxicated mice, *M. pruriens* and levodopa improved signs of Parkinson’s disease [82]. The n-propanol extract of boiled and fermented seeds of *M. pruriens* can offer greater neuroprotection against dopaminergic neurons than fresh seeds, as observed in a Parkinson’s disease rat model [83]. In other studies, the seed extract of *M. pruriens* enhanced learning and memory [84]. It increased the number of surviving neurons in the CA1 and CA3 regions of the hippocampus. Liver and kidney function tests revealed good recovery after long-term treatment with *M. pruriens* [85]. *M. pruriens* prevents depression-like behaviors by decreasing oxidative stress and lipopolysaccharide effects [86]. It has also been reported to have antiepileptic, antidiabetic, and antineoplastic effects [87].

The bioactive molecules of *M. pruriens* can be useful in the management of pulmonary hypertension [88]. The antivenom activity of this plant has also been investigated [89]. Levodopa is also responsible for other pharmacological effects of plants, such as antidiabetic, anti-inflammatory, neuroprotective, and antioxidant effects [90]. It is well known for its aphrodisiac properties, as it can increase sperm count and testosterone levels. Its use in infertility was confirmed in an animal model study in albino mice [91]. The seed extract of Mucuna decreased damage to tissue structure and sperm parameters in sodium arsenite-treated rats [92]. Mucuna contains levodopa, which helps in penile erection [93]. Its seeds have shown promising results in assays of total phenolic content, ferric reducing antioxidant power, and deoxyribonucleic acid damage protection [94]. A study from Thailand reported that *M. pruriens* seed extract prevented testicular apoptosis by affecting caspase, proliferating cell nuclear antigen, and dopamine receptor D2 protein expression [95]. During cell metabolism, free radicals are produced from one or more unpaired electrons. The free radical-scavenging properties of polyphenols make them important phytochemicals for managing oxidative stress. Flavonoids are simple phenolic compounds with antioxidant properties [96]. In experimental rodents, the ethanol extract of *M. pruriens* leaves was shown to exert anti-inflammatory activity by inhibiting oxidative stress, proinflammatory cytokines, and lysosomal membrane instability [97].

### Effects of *M. pruriens* in cerebral ischemia

*M. pruriens* is helpful against global cerebral ischemia/reperfusion injury-induced oxidative stress in rats, and it is likely to be useful in ischemic treatment. The methanolic extract of *M. pruriens* was shown to reduce the incidence of ischemic brain injury-induced by occlusion of the common carotid artery in a rat model [98]. Nayak et al. [23] reported that *M. pruriens* ameliorated decreased activity observed in ischemic animals. This treatment decreased the elevated LPO level and increased the levels of SOD, CAT, GSH, and total thiols in the ischemic group, suggesting a protective effect of the extract against oxidative stress. In this study, histopathological observation of hippocampal CA1 neurons revealed a decrease in the number of viable neurons in the stress-induced group. Animals in the *M. pruriens* extract-pretreated ischemic group presented a dose-dependent decrease in neuronal damage, indicating neuroprotective activity. The observed impaired motor coordination in the aluminum fluoride-treated animals was minimal in the extract-treated groups. *M. pruriens* can alter neurotransmitter levels and the balance of excitatory and inhibitory inputs to Purkinje cells of the cerebellum.

**Figure 2:**
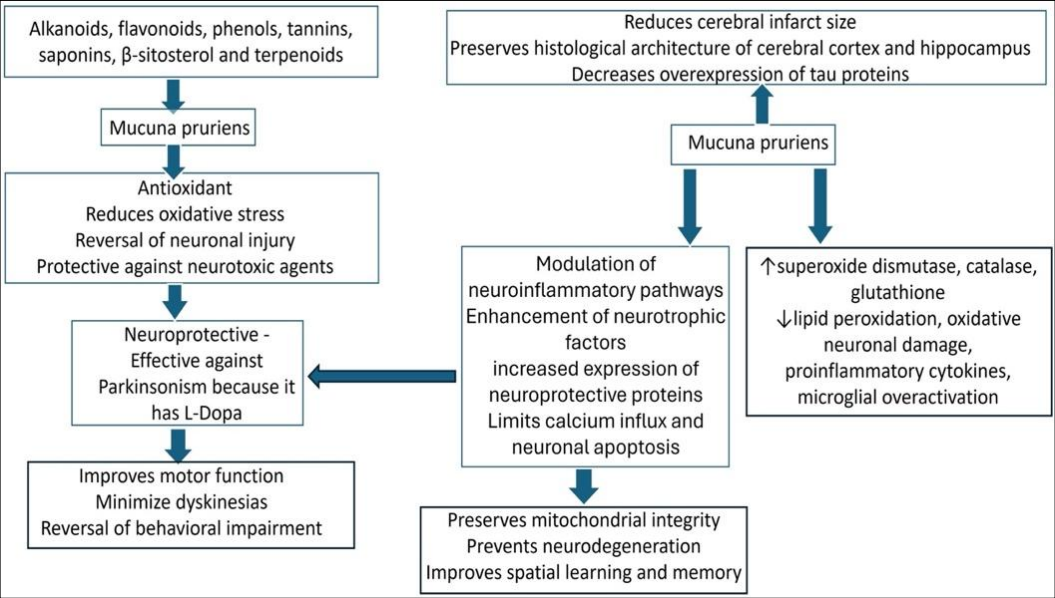
Schematic representation of the mechanism of action and neuroprotective role of *Mucuna pruriens* [21–26, 45, 70–87, 91, 94, 97].

*M. pruriens* offered neuroprotection in a global cerebral ischemia animal model induced by occlusion of the bilateral common carotid artery [23]. This was evident in histopathological and biochemical analyses. It was reported that treatment with methanolic extracts of *M. pruriens* seeds improved locomotion, coordination, and spatial learning. It also reduced the cerebral infarct area, indicating its therapeutic efficacy in treating cerebral ischemia [99]. This is due to its antioxidant properties, which increase the levels of antioxidant enzymes and reduce lipid peroxidation [100]. A methanolic extract of *M. pruriens* significantly increased the levels of SOD, CAT, GPx, and glutathione reductase in high-fat diet-fed rats [101]. It also elevates nonenzymatic antioxidants, such as glutathione, further indicating a reduction in oxidative stress. This study also revealed a decrease in thiobarbituric acid-reactive substances, which are markers of lipid peroxidation, and this finding was statistically significant (*p* < 0.05). Krishna and Sundararajan [102] compared the effects of different doses of the methanolic extract of *M. pruriens* and reported that higher doses significantly increased the ferric reducing ability of plasma and CAT levels (*p* < 0.05). It also reduced thiobarbituric acid-reactive substances levels, suggesting a dose-dependent antioxidant effect of *M. pruriens*. It has been reported that prophylactic treatment with *M. pruriens* and its bioactive molecule, β-sitosterol, downregulates the expression of the N-methyl-D-aspartate receptor and tau protein genes in the ischemic brain, which are usually upregulated during cerebral ischemia [98]. The comprehensive mechanism of action and neuroprotective role of *M. pruriens* are schematically represented in Figure 2 [21–26, 45, 70–87, 91, 94, 97]. *M. pruriens* has potential for preischemic conditioning, which is similar to ischemic preconditioning. It is hypothesized that pretreatment with *M. pruriens* may enhance brain resilience.

### Challenges and limitations of the use of *M. pruriens* as neuroprotective agents

*M. pruriens* has been proven to be neuroprotective in ischemia-induced animal models, but its efficacy can vary. Its effect is due to antioxidative and anti-inflammatory properties; however, this effect may not be sufficient in all cases of cerebral ischemia [23, 99]. In a spinal cord injury animal model, no statistically significant beneficial effects were observed, suggesting variable efficacy across different types of neuronal injury [21]. Another challenge is its bioavailability, as the blood–brain barrier can limit delivery of Mucuna compounds to the brain, potentially decreasing their efficacy in treating cerebral ischemia. The dosage and route of administration of *M. pruriens* need to be standardized to ensure that active compounds reach brain tissue.

### Translational barriers

The application of translational research and results from animal model studies to human clinical trials and experimental setups may be uncertain. Variability among individual subjects and efficacy across various species present significant challenges. There is a need for better guidelines to achieve significant therapeutic outcomes [23, 98, 99]. *M. pruriens* is effective as a potential neuroprotective agent because of its relatively high content of levodopa, which is a precursor of dopamine, and other bioactive compounds, such as tyrosine [86]. Hence, *M. pruriens* can be contraindicated in patients with comorbidities such as Parkinson’s disease, where it is already used as treatment, because it can cause toxicity [103]. The quantity of bioactive compounds in *M. pruriens* can vary depending on the extract and preparation methods, which can lead to variability in efficacy [90]. Long-term use of *M. pruriens* can cause levodopa-like dyskinesias [104]. Hence, understanding these side effects in future studies is crucial.

### Regulatory guidelines

It has also been reported that unprocessed *M. pruriens* may contain antinutritional components and could be toxic to ruminants [76]. In some regions, such as Japan, *M. pruriens* products are not pharmaceuticals and can be sold online, and permission from food regulatory authorities is not needed. However, specific legal requirements apply, and these products should not be advertised inappropriately, as this may lead to incorrect messaging and misuse [105].

### Future implications

Although *M. pruriens* effectively reduces ischemic damage to the brain, as evidenced by histopathological analysis, the degree of neuroprotection can vary. Further research is essential for understanding the potential factors responsible for this variability [98]. The present evidence comes from preclinical animal model studies, and human clinical trials are essential for establishing standardized therapeutic guidelines. More research on *M. pruriens* is needed to study its comprehensive effects in treating cerebral ischemia, as its etiopathogenesis is complex, involving oxidative stress, neuroinflammation, and neuronal death. The future implications include studying it as an adjuvant and as a combination therapy along with other drugs. Nanoformulations of *M. pruriens*, blood–brain barrier-permeable phytochemical fractions, standardized extraction methods, and combination therapy with reperfusion drugs should be studied in future research.

## CONCLUSION

The present review highlights that cerebral ischemia is a multifactorial neurological disorder characterized by oxidative stress, neuroinflammation, excitotoxicity, mitochondrial dysfunction, and apoptosis, ultimately leading to neuronal damage and functional impairment. Evidence from animal model studies consistently demonstrates that *M. pruriens* exerts significant neuroprotective effects by enhancing endogenous antioxidant defenses, including SOD, CAT, and GPx, while reducing lipid peroxidation markers, including MDA. Histopathological findings reveal preservation of neuronal integrity, particularly in the hippocampal regions, and a reduction in infarct size. Behavioral assessments further confirm improvements in locomotor activity, motor coordination, and cognitive performance, indicating a functional recovery following treatment with *M. pruriens*.

From a practical perspective, the findings of this review suggest that *M. pruriens* has potential as an adjunct therapeutic agent in the management of cerebral ischemia. Its rich phytochemical composition, including levodopa, flavonoids, and phenolic compounds, enables it to act through multiple mechanisms, such as free-radical-scavenging, modulation of neurotransmitter balance, and regulation of inflammatory pathways. These multimodal actions make it a promising candidate for integration into complementary and alternative therapeutic strategies, particularly in conditions where conventional treatments are limited or associated with adverse effects.

A major strength of this review lies in its comprehensive synthesis of animal model–based evidence, integrating biochemical, histopathological, and behavioral outcomes to provide a holistic understanding of the neuroprotective role of *M. pruriens*. The inclusion of multiple experimental models and comparative analyses enhances the reliability of the conclusions and supports the translational relevance of the findings.

However, several limitations must be acknowledged. The variability in experimental design, dosages, extraction methods, and treatment durations across studies limits the consistency and comparability of results. In addition, differences in animal species and models restrict the direct extrapolation of findings to human clinical conditions. The lack of standardized formulations and insufficient clinical trials further constrain the therapeutic application of *M. pruriens* in routine medical practice.

In conclusion, *M. pruriens* demonstrates promising neuroprotective potential in cerebral ischemia through its antioxidant, anti-inflammatory, and antiapoptotic properties. While preclinical findings are encouraging, there is a critical need for well-designed, standardized, and large-scale studies to validate its efficacy and safety. Future research focusing on dose-optimization, bioavailability, and clinical translation will be essential to establish *M. pruriens* as a reliable neuroprotective agent in ischemic stroke management.

## DATA AVAILABILITY

The supplementary data can be made available from the corresponding author upon request.

## AUTHORS’ CONTRIBUTIONS

VSN, KSRP, and SSN: Performed data acquisition and analysis. VSN, SSN, BVM, and SS: Wrote the manuscript. KSRP, SS, and MT: Manuscript review. All authors have read, reviewed, and approved the final version of the manuscript.

## References

[1] Zhu W, He X, Huang D, Jiang Y, Hong W, Ke S (2025). Global and regional burden of ischemic stroke disease from 1990 to 2021: An age-period-cohort analysis. Transl Stroke Res.

[2] Hou S, Zhang Y, Xia Y, Liu Y, Deng X, Wang W (2024). Global, regional, and national epidemiology of ischemic stroke from 1990 to 2021. Eur J Neurol.

[3] Neumar RW (2000). Molecular mechanisms of ischemic neuronal injury. Ann Emerg Med.

[4] Ozbal S, Erbil G, Kocdor H, Tugyan K, Pekcetin C, Ozogul C (2008). The effects of selenium against cerebral ischemia–reperfusion injury in rats. Neurosci Lett.

[5] Broughton BR, Reutens DC, Sobey CG (2009). Apoptotic mechanisms after cerebral ischemia. Stroke.

[6] Malhotra K, Liebeskind DS (2020). Collaterals in ischemic stroke. Brain Hemorrhages.

[7] Wu L, Xiong X, Wu X, Ye Y, Jian Z, Zhi Z (2020). Targeting oxidative stress and inflammation to prevent ischemia–reperfusion injury. Front Mol Neurosci.

[8] Cui H, Kong Y, Zhang H (2012). Oxidative stress, mitochondrial dysfunction, and aging. J Signal Transduct.

[9] Zengin G, Terzić M, Abul N, Gülçin İ, Koyuncu İ, Basarali MK (2025). A multidimensional study for design functional foods: Chemical profiling, antioxidant potential, enzyme inhibition, and cytotoxic effects of Alkanna tubulosa extracts. Food Biosci.

[10] Sims NR, Anderson MF, Hobbs LM, Kong JY, Phillips S, Powell JA (2000). Impairment of brain mitochondrial function by hydrogen peroxide. Brain Res Mol Brain Res.

[11] Smith WS (2004). Pathophysiology of focal cerebral ischemia: A therapeutic perspective. J Vasc Interv Radiol.

[12] Yapıcı I, Altay A, Ozturk Sarikaya SB, Korkmaz M, Atila A, Gulçin İ (2021). *In vitro* antioxidant and cytotoxic activities of extracts of endemic Tanacetum erzincanense together with phenolic content by LC-ESI-QTOF-MS. Chem Biodivers.

[13] Struneck A, Patocka J, Shapiro P, Atwood D (2002). Aluminofluoride complexes: A useful tool in laboratory investigations, but a hidden danger for living organisms?. Fundamental research, material science and catalysis.

[14] Chirumari K, Reddy PK (2007). Dose-dependent effects of fluoride on neurochemical milieu in the hippocampus and neocortex of rat brain. Fluoride.

[15] Hu X, Guo Z, Shi Z, Zhen P, Zhou M (2025). Morphological changes in CA3 pyramidal neurons after transient global ischemia. Neuroreport.

[16] Li H, Xue X, Li L, Li Y, Wang Y, Huang T (2020). Aluminum-induced synaptic plasticity impairment via PI3K-Akt-mTOR signaling pathway. Neurotox Res.

[17] Li H, Xue X, Li Z, Pan B, Hao Y, Niu Q (2020). Aluminiuium-induced synaptic plasticity injury via the PHF8-H3K9me2-BDNF signaling pathway. Chemosphere.

[18] Yang L, Qian X, Jiang H, Xie C (2025). Inhibition of SIRT1/PGC-1αaxis exacerbates fluorine and aluminium induced neurotoxicity via Drp1-dependent aggravated mitochondrial fission. Mol Neurobiol.

[19] Xin W, Pan Y, Wei W, Tatenhorst L, Graf I, Popa-Wagner A (2023). Preconditioned extracellular vesicles from hypoxic microglia reduce poststroke AQP4 depolarization, disturbed cerebrospinal fluid flow, astrogliosis, and neuroinflammation. Theranostics.

[20] Jain M, Das S, Lu PPY, Virmani G, Soman S, Thumu SCR (2021). SRF is required for maintenance of astrocytes in non-reactive state in the mammalian brain. eNeuro.

[21] Chandran P, Chandramohan K, Iyer K, Michael FM, Seppan P, Venkatachalam S (2022). Beneficial effects of ethanolic extract of the medicinal herb *Mucuna pruriens* against oxidative stress and inflammation might be limited in contusive spinal cord injury. Biomed Pharmacol J.

[22] Bhosle PV, Wadher SJ (2025). Evaluation of *Mucuna pruriens* extract as a potential treatment for Huntington's disease: Antioxidant and anti-inflammatory mechanisms in rat models. J Neurosci Rural Pract.

[23] Nayak VS, Kumar N, D'Souza AS, Nayak SS, Cheruku SP, Pai KSR (2017). The effects of *Mucuna pruriens* extract on histopathological and biochemical features in the rat model of ischemia. Neuroreport.

[24] Dogra N, Nagpal D, Aeri V, Ahmad S, Pande Katare D (2021). Evaluating the synergistic effect of Mucuna prurines extract and sesame oil against the Parkinson's disease zebrafish model: *In vivo*/*in silico* approach. All Life.

[25] Yadav SK, Prakash J, Chouhan S, Westfall S, Verma M, Singh TD (2014). Comparison of the neuroprotective potential of *Mucuna pruriens* seed extract with estrogen in 1-methyl-4-phenyl-1,2,3,6-tetrahydropyridine (MPTP)-induced PD mice model. Neurochem Int.

[26] Manyam BV, Dhanasekaran M, Hare TA (2004). Neuroprotective effects of the antiparkinson drug *Mucuna pruriens*. Phytother Res.

[27] Chukanova AS, Chukanova EI, Nadareishvili GG, Gulieva MS, Gusev EI (2017). Patogeneticheskie aspekty formirovaniia ostroĭfokal'noĭishemii golovnogo mozga [Pathogenetic aspects of the development of acute focal cerebral ischemia. Zh Nevrol Psikhiatr Im S S Korsakova.

[28] Schaller BJ (2007). Influence of age on stroke and preconditioning-induced ischemic tolerance in the brain. Exp Neurol.

[29] Quillinan N, Herson PS, Traystman RJ (2016). Neuropathophysiology of brain injury. Anesthesiol Clin.

[30] Li Z, Li M, Fang Z, Wang H (2025). Immunological mechanisms and therapeutic strategies in cerebral ischemia–reperfusion injury: From inflammatory response to neurorepair. Int J Mol Sci.

[31] Mader MM, Heimann A, Kempski O, Wöbker G, Alessandri B (2022). Multiparametric monitoring of early pathophysiological changes in a porcine model of sequential focal and global cerebral ischemia. World Neurosurg.

[32] Ister R, Pongrac M, DobrivojevićRadmilović M (2024). Optimization of the longa middle cerebral artery occlusion method for complete reperfusion. J Vis Exp.

[33] Ahad MA, Kumaran KR, Ning T, Mansor NI, Effendy MA, Damodaran T (2020). Insights into the neuropathology of cerebral ischemia and its mechanisms. Rev Neurosci.

[34] Pan J, Konstas AA, Bateman B, Ortolano GA, Pile-Spellman J (2007). Reperfusion injury following cerebral ischemia: Pathophysiology, MR imaging, and potential therapies. Neuroradiology.

[35] Kahl A, Stepanova A, Konrad C, Anderson C, Manfredi G, Zhou P (2018). Critical role of flavin and glutathione in complex I-mediated bioenergetic failure in brain ischemia/reperfusion injury. Stroke.

[36] Luo T, Park Y, Sun X, Liu C, Hu B (2013). Protein misfolding, aggregation, and autophagy after brain ischemia. Transl Stroke Res.

[37] Zhang Y, Liu L, Hou X, Zhang Z, Zhou X, Gao W (2023). Role of autophagy mediated by AMPK/DDiT4/mTOR axis in HT22 cells under oxygen and glucose deprivation/reoxygenation. ACS Omega.

[38] Wang L, Zhang X, Xiong X, Zhu H, Chen R, Zhang S (2022). Nrf2 regulates oxidative stress and its role in cerebral ischemic stroke. Antioxidants (Basel).

[39] Li Y, Jiang J, Zhuo Y, Li J, Li Y, Xia Y (2025). IGF2BP1 exacerbates neuroinflammation and cerebral ischemia/reperfusion injury by regulating neuronal ferroptosis and microglial polarization. Biochim Biophys Acta Mol Basis Dis.

[40] Gupta YK, Briyal S (2004). Animal models of cerebral ischemia for evaluation of drugs. Indian J Physiol Pharmacol.

[41] Li T, Xiam J, Dong Y, Chen J, Ren M (2026). Controlled intracranial pressure elevation via cerebrospinal fluid infusion: A novel hemostatic hypothesis for hematoma expansion in traumatic brain contusion. Medical Hypotheses.

[42] Basilio AV, Zeng D, Pichay LA, Ateshian GA, Xu P, Maas SA (2024). Simulating cerebral edema and ischemia after traumatic acute subdural hematoma using triphasic swelling biomechanics. Ann Biomed Eng.

[43] Donnelly J, Czosnyka M, Harland S, Varsos GV, Cardim D, Robba C (2018). Increased ICP and its cerebral haemodynamic sequelae. Acta Neurochir Suppl.

[44] Lafrenaye AD, Krahe TE, Povlishock JT (2014). Moderately elevated intracranial pressure after diffuse traumatic brain injury is associated with exacerbated neuronal pathology and behavioral morbidity in the rat. J Cereb Blood Flow Metab.

[45] Verma R, Bisen P (2021). *Mucuna pruriens* - a wonder herb for degenerative disorders. In:An introduction to medicinal herbs. Chapter 8. Nova Science Publisher.

[46] Jin X, Jing W, Fengxia Y, Zhang Z, Fengjun L, Jing S (2007). Effect of intracranial hypertension on cerebral hemorrhage induced autonomic nerve imbalance. Neural Reg Research.

[47] Abdou H, Treffalls R, Jodlowski G, Elansary N, Ptak T, Walker PF (2025). The influence of hemorrhagic shock on brain perfusion in a swine model of raised intracranial pressure. Eur J Trauma Emerg Surg.

[48] Hamilton DR, Sargsyan AE, Melton SL, Garcia KM, Oddo B, Kwon DS (2011). Sonography for determining the optic nerve sheath diameter with increasing intracranial pressure in a porcine model. J Ultrasound Med.

[49] Calloni RL, Winkler BC, Ricci G, Poletto MG, Homero WM, Serafini EP (2010). Transient middle cerebral artery occlusion in rats as an experimental model of brain ischemia. Acta Cir Bras.

[50] Vidale S, Consoli A, Arnaboldi M, Consoli D (2017). Postischemic inflammation in acute stroke. J Clin Neurol.

[51] Gerriets T, Stolz E, Walberer M, Müller C, Rottger C, Kluge A (2004). Complications and pitfalls in rat stroke models for middle cerebral artery occlusion: A comparison between the suture and the macrosphere model using magnetic resonance angiography. Stroke.

[52] Nagel S, Papadakis M, Chen R, Hoyte LC, Brooks KJ, Gallichan D (2011). Neuroprotection by dimethyloxalylglycine following permanent and transient focal cerebral ischemia in rats. J Cereb Blood Flow Metab.

[53] Nour M, Scalzo F, Liebeskind DS (2013). Ischemia-reperfusion injury in stroke. Intervent Neurol.

[54] McCulloch J, Ozyurt E, Park CK, Nehls DG, Teasdale GM, Graham DI, Baethmann A, Kempski O, Schürer L (1993). Glutamate receptor antagonists in experimental focal cerebral ischaemia. Mechanisms of secondary brain damage. Acta Neurochirurgica.

[55] Zhao LQ, Parikh A, Xiong YX, Ye QY, Ying-Guo, Zhou XF (2022). Neuroprotection of oral edaravone on middle cerebral artery occlusion in rats. Neurotox Res.

[56] Rahmati-Dehkordi F, Khanifar H, Zare-Hoseinabadi A, Dadgostar E, Jafarpour H, Aschner M (2024). Potential of edaravone dexborneol in the treatment of cerebral ischemia: Focus on cell death-related signaling pathways. Mol Biol Rep.

[57] Horn J, de Haan RJ, Vermeulen M, Luiten PG, Limburg M (2001). Nimodipine in animal model experiments of focal cerebral ischemia: A systematic review. Stroke.

[58] Tang XN, Wang Q, Koike MA, Cheng D, Goris ML, Blankenberg FG (2007). Monitoring the protective effects of minocycline treatment with radiolabeled annexin V in an experimental model of focal cerebral ischemia. J Nucl Med.

[59] Song X, Jensen MØ, Jogini V, Stein RA, Lee CH, Mchaourab HS (2018). Mechanism of NMDA receptor channel block by MK-801 and memantine. Nature.

[60] Zhang XY, Ho HL, Feng ZP, Sun HS (2026). Neuroprotective effects of carvacrol in cerebral ischemia and hypoxia. International Journal of Drug Discovery and Pharmacology.

[61] Thaysen M, Westi E, Clarkson AN, Wellendorph P, Kristensen M (2024). Rodent ischemic stroke models and their relevance in preclinical research. Neuroprotection.

[62] Rodriguez-Amaya DB (2003). Food carotenoids: Analysis, composition and alterations during storage and processing of foods. Forum Nutr.

[63] Yadav NP, Dixit VK (2008). Recent approaches in herbal drug standardization. Int J Integr Biol.

[64] Parivuguna V, Gnanaprabhal R, Dhanabalan R, Doss A (2008). Antimicrobial properties and phytochemical constituents of Rheo discolour Hance. Ethnobotanical Leaflets.

[65] Beauchamp GK, Keast RS, Morel D, Lin J, Pika J, Han Q (2005). Phytochemistry: Ibuprofen-like activity in extra-virgin olive oil. Nature.

[66] Smeriglio A, Barreca D, Bellocco E, Trombetta D (2017). Proanthocyanidins and hydrolysable tannins: Occurrence, dietary intake and pharmacological effects. Br J Pharmacol.

[67] Das S, Rajeswari VD, Venkatraman G, Ramanathan G Phytochemicals in Parkinson's disease: A pathway to neuroprotection and personalized medicine. Cell Biochem Biophys.

[68] Alam N, Hossain M, Mottalib MA, Sulaiman SA, Gan SH, Khalil MI (2012). Methanolic extracts of Withania somnifera leaves, fruits and roots possess antioxidant properties and antibacterial activities. BMC Complement Altern Med.

[69] Narayanasamy A, Kanagaraja A, Thirumavalavan M, Sakthivelu M, Pachaiappan R (2026). Evaluation of antioxidant activities of bioactive peptides extracted from Curcuma longa and Curcuma caesia from South-eastern and North-Eastern India. Probiotics Antimicro Prot.

[70] Osuntokun OS, Olayiwola G, Oriare AK, Oyedokun SO, Abayomi TA, Tokunbo OS (2022). *Mucuna pruriens* seed protects the hippocampal neurons and abrogates seizure indices in chemically-convulsed mice: Evidence of the Nrf2 expression defense pathway. J Chem Neuroanat.

[71] Lampariello LR, Cortelazzo A, Guerranti R, Sticozzi C, Valacchi G (2012). The magic velvet bean of *Mucuna pruriens*. J Tradit Complement Med.

[72] Hammoud F, Ismail A, Zaher R, El Majzoub R, Abou-Abbas L (2025). *Mucuna pruriens* treatment for Parkinson's disease: A systematic review of clinical trials. Parkinson's Dis.

[73] Dendup T, Prachyawarakorn V, Pansanit A, Mahidol C, Ruchirawat S, Kittakoop P (2014). α-Glucosidase inhibitory activities of isoflavanones, isoflavones, and pterocarpans from *Mucuna pruriens*. Planta Med.

[74] Rai SN, Chaturvedi VK, Singh P, Singh BK, Singh MP (2020). *Mucuna pruriens* in Parkinson's and in some other diseases: Recent advancement and future prospective. 3 Biotech.

[75] Aslam S, Rafiq A, Ahmad M, Naqvi SAR, Al-Huqail AA, Zia-Ul-Haq M, Al-Huqail AA, Riaz M, Farooq Gohar U (2023). Cowhage. Essentials of medicinal and aromatic crops.

[76] Pathania R, Chawla P, Khan H, Kaushik R, Khan MA (2020). An assessment of potential nutritive and medicinal properties of *Mucuna pruriens*: A natural food legume. 3 Biotech.

[77] Deli M, Nguimbou RM, Djantou EB, Tatsadjieu Ngoune L, Njintang Yanou N, Murthy HN, Paek KY (2021). Bioactive compounds of velvet bean (*Mucuna pruriens* L.). seeds. Bioactive compounds in underutilized vegetables and legumes. Reference Series in Phytochemistry.

[78] Yadav M, Upadhyay P, Purohit P, Pandey B, Shah H (2017). Phytochemistry and pharmacological activity of *Mucuna pruriens*: A review. International Journal of Green Pharmacy.

[79] Jimoh MA, Idris OA, Jimoh MO (2020). Cytotoxicity, phytochemical, antiparasitic screening, and antioxidant activities of *Mucuna pruriens* (Fabaceae). Plants.

[80] Sruthi D, Jayabaskaran C (2024). Performance of different solvents and extraction methods on therapeutic potential of *Mucuna pruriens* (L.). DC. and chemical profiling of screened extract with chromatography-mass spectrometry approach. Curr Res Cmpl Alt Med.

[81] Mata-Bermudez A, Diaz-Ruiz A, Silva-García LR, Gines-Francisco EM, Noriega-Navarro R, Rios C (2024). *Mucuna pruriens*, a possible treatment for depressive disorders. Neurol Int.

[82] Zaigham SB, Paeng DG (2024). Effects of *Mucuna pruriens* (L.). DC. and levodopa in improving Parkinson's disease in rotenone intoxicated mice. Curr Issues Mol Biol.

[83] Adi YK, Widayanti R, Pangestiningsih TW (2018). n-Propanol extract of boiled and fermented koro benguk (*Mucuna pruriens* seed) shows a neuroprotective effect in paraquat dichloride-induced Parkinson's disease rat model. Vet World.

[84] Osuntokun OS, Olayiwola G, Oriare AK, Odeniran HT, Ayoka AO (2021). The activities of the central nervous system following ethyl acetate extract of *Mucuna pruriens* seed administration in male BALB/c mice. Nig J Neurosci.

[85] Concessao P, Bairy LK, Raghavendra AP (2020). Protective effect of *Mucuna pruriens* against arsenic-induced liver and kidney dysfunction and neurobehavioral alterations in rats. Vet World.

[86] Mata-Bermudez A, Trejo-Chávez R, Martínez-Vargas M, Pérez-Arredondo A, de Los Ángeles, Martínez-Cardenas M (2024). Effect of *Mucuna pruriens* seed extract on depression-like behavior derived from mild traumatic brain injury in rats. Biomedicine (Taipei).

[87] Sathiyanarayanan L, Arulmozhi S (2007). *Mucuna pruriens*: A comprehensive review. Pharmacogn Rev.

[88] Bhosle S, Bagali S, Parvatikar PP, Das KK (2024). Effect of bioactive compounds of *Mucuna pruriens* on proteins of Wnt/βcatenin pathway in pulmonary hypertension by *in silico* approach. In Silico Pharmacol.

[89] Guerranti R, Ogueli IG, Bertocci E, Muzzi C, Aguiyi JC, Cianti R (2008). Proteomic analysis of the pathophysiological process involved in the antisnake venom effect of *Mucuna pruriens* extract. Proteomics.

[90] Misra L, Wagner H (2007). Extraction of bioactive principles from *Mucuna pruriens* seeds. Indian J Biochem Biophys.

[91] Arif M, Alam M, Malhi SM, Azeem Z, Sherwani B, Asghar MN (2024). *In vivo* evaluation of medicinal effects of Myristica fragrans, Cinnamomum zeylanicum, *Mucuna pruriens* on male murine fertility. Pak J Pharm Sci.

[92] Concessao PL, Bairy KL, Raghavendra AP (2023). Ameliorating effect of *Mucuna pruriens* seed extract on sodium arsenite-induced testicular toxicity and hepato-renal histopathology in rats. Vet World.

[93] Wattanawiggan R, Chansakaow S, Jantrawut P, Panraksa P, Jiaranaikulwanitch J, Udomsom S (2024). Design and optimization of 3D-printed tablets containing Mucuna extracts for erectile dysfunction management: A DoE-guided study. Plants (Basel).

[94] Kumbhare SD, Ukey SS, Gogle DP (2023). Antioxidant activity of Flemingia praecox and *Mucuna pruriens* and their implications for male fertility improvement. Sci Rep.

[95] Samrid R, Taoto C, Wu A, Sawatpanich T, Phunchago N, Uabundit N (2023). Protective effect of *Mucuna pruriens* (L.). DC. var. pruriens seed extract on apoptotic germ cells in ethanolic male rats. Braz J Biol.

[96] Beta T, Nam S, Dexter JE, Sapirstein HD (2005). Phenolic content and antioxidant activity of pearled wheat and roller-milled fractions. Cereal Chem.

[97] Alabi AO, Ogunjimi LO, Murtala AA, Kasumu EO, Oyinloye EO, Shofoyeke AM (2024). Sub-acute toxicity, antinociceptive and anti-inflammatory effects of *Mucuna pruriens* L. leaves in experimental rodents. J Ethnopharmacol.

[98] Parvatikar PP, Patil SM, Patil BS, Reddy RC, Bagoji I, Kotennavar MS (2023). Effect of *Mucuna pruriens* on brain NMDA receptor and tau protein gene expression in cerebral ischemic rats. Front Physiol.

[99] Nayak VS, Pai KSR, Nayak SS, Kumar N, Bangera H (2021). Effect of *Mucuna pruriens* (Linn.). on global cerebral ischemia-induced motor incoordination. Trop J Pharma Res.

[100] Oyinloye OE, Murtala AA, Oladoja FA, Okunye OL, Aderinola AA, Kasumu EO (2023). Evaluation of phytochemical constituents, total phenolic contents and antioxidant activities of *Mucuna pruriens* fractions leaves. J Phytomed Ther.

[101] Kumar DS, Muthu AK, Smith AA, Manavalan R (2011). *In vivo* antioxidant and lipid peroxidation effect of various extracts of whole plant of *Mucuna pruriens* (Linn) in rat fed with high-fat diet. Asian J Chem.

[102] Krishna RG, Sundararajan R (2019). Screening of antioxidant activity of *Mucuna pruriens* by *in vivo* model. Int J Res Pharm Sci.

[103] Kumar N, Singh SK, Lal RK, Dhawan SS (2024). An insight into dietetic and nutraceutical properties of underutilized legume: *Mucuna pruriens* (L.) DC. J Food Comp Anal.

[104] Panova AS, Dergachev DS, Subotyalov MA, Dergachev VD (2020). Review of *Mucuna pruriens* L. therapeutic potential for Parkinson's disease. Meditsinskiy Sovet.

[105] Sato K, Hida A, Niimi Y, Iwata A, Iwatsubo T (2023). Survey on the current advertising and sales of *Mucuna pruriens* in consumer-to-consumer internet trading in Japan. Yakugaku Zasshi.

